# Insight Into the Interaction Between RNA Polymerase and VPg for Murine Norovirus Replication

**DOI:** 10.3389/fmicb.2018.01466

**Published:** 2018-07-03

**Authors:** Ji-Hye Lee, Beom Seok Park, Intekhab Alam, Kang R. Han, Scott B. Biering, Soo J. Kim, Jayoung Choi, Jong H. Seok, Mi S. Chung, Ho M. Kim, Seungmin Hwang, Kyung H. Kim

**Affiliations:** ^1^Department of Biotechnology & Bioinformatics, Korea University, Sejong, South Korea; ^2^Department of Biomedical Laboratory Science, College of Health Science, Eulji University, Gyeonggi-do, South Korea; ^3^Committee on Microbiology, The University of Chicago, Chicago, IL, United States; ^4^Graduate School of Medical Science and Engineering, College of Life Science and Bioengineering, Korea Advanced Institute of Science and Technology, Daejeon, South Korea; ^5^Department of Pathology, The University of Chicago, Chicago, IL, United States; ^6^Department of Food and Nutrition, Duksung Women’s University, Seoul, South Korea

**Keywords:** norovirus, RNA-dependent RNA polymerase, VPg, interaction, viral replication

## Abstract

Norovirus (NoV) is a leading cause of epidemic acute non-bacterial gastroenteritis, and replicates through virion protein genome-linked (VPg)-primed or *de novo* RNA synthesis by RNA-dependent RNA polymerase (RdRp). VPg is a multifunctional protein that plays crucial roles in viral protein translation and genome replication. However, the interaction between the RdRp and this multifunctional VPg in NoV replication has been unknown. In this study, VPg derived from murine NoV (MNV) was found to mediate the formation of higher-order multimers or tubular fibrils of MNV RdRp, which led to significantly enhanced polymerase activity *in vitro*. The replication of MNV mutants containing a VPg-binding defective RdRp, based on the crystal structure of an RdRp-VPg(1-73) complex, was completely blocked in a cell culture system. Our data suggest that the interaction between RdRp and VPg plays a crucial role in the multimerization-mediated RdRp activity *in vivo* and consequently in MNV replication, which can provide a new target of therapeutic intervention for NoV outbreaks.

## Introduction

Human norovirus (HuNV) is currently a leading cause of acute gastroenteritis in adults and predicted to become the predominant cause of diarrhea in all age groups worldwide (Patel et al., 2008; Bok and Green, 2012). HuNV can establish chronic infection and becomes life-threatening in immunocompromised patients (Bok and Green, 2012). However, no effective antiviral or vaccine against HuNV are available yet, mainly due to the lack of an efficient *in vitro* and *in vivo* infection system of HuNV. The recent developments of immortalized B cell lines, the stem-cell-derived organoid system, and BALB/c Rag-γc-deficient mice for HuNV infection provide new models to study HuNV (Taube et al., 2013; Jones et al., 2014; Ettayebi et al., 2016). Since its isolation from immunocompromised mice (Wobus et al., 2004), murine NoV (MNV) has been serving as an effective surrogate model (Karst et al., 2003; Wobus et al., 2006) to elucidate the molecular mechanism of NoV replication and pathogenesis.

Norovirus (NoV) is a positive-sense single-stranded RNA (+RNA) virus belonging to the *Caliciviridae* family. The ∼7.6 kb +RNA genome of NoV contains three open reading frames (ORFs) (Hardy, 2005), with an additional ORF4 for MNV (McFadden et al., 2011). ORF1 encodes a polyprotein that is cleaved into non-structural proteins, including virion protein genome-linked (VPg) and RNA-dependent RNA polymerase (RdRp) (Sosnovtsev et al., 2006). RdRp amplifies the viral genome using ribonucleotide triphosphates (rNTPs) as substrates in low-fidelity due to the lack of an effective proofreading mechanism. The initiation of RNA synthesis by RdRp on an RNA template can be either primer-dependent or -independent in *Caliciviridae*, *Picornaviridae*, and *Potyviridae* (Green, 2007; Goodfellow, 2011; Jiang and Laliberte, 2011). In the former case, VPg serves as a primer for these viruses, providing free hydroxyl groups from a tyrosine or serine residue. VPg covalently attached to the 5′ end of viral RNAs plays crucial roles in viral protein translation and genome replication (Burroughs and Brown, 1978; Herbert et al., 1997; Goodfellow, 2011; Jiang and Laliberte, 2011).

VPg is an intrinsically disordered molten globule-like protein with multiple functions. It is highly diverse in sequence and size, ranging from 2 to 90 kDa, of which the largest belongs to *Birnaviridae* possessing bisegmented dsRNAs with its RdRp linked as VPg (Calvert et al., 1991). The genomic RNA of feline calicivirus, a member of *Caliciviridae*, is not infectious after proteolytic cleavage of the VPg (Herbert et al., 1997). VPg was shown to bind to its cognate RdRp in enterovirus71 (EV71), foot and mouth disease virus (FMDV), and coxsackievirus (CV) (Ferrer-Orta et al., 2006; Gruez et al., 2008; Chen et al., 2013), with which the VPg of caliviruses shares no sequence homology. No caliciviral RdRp-VPg complex structure is yet available and the role of the RdRp–VPg interaction in NoV replication remains unknown.

In this study, we characterized some biochemical and biophysical properties the MNV RdRp-VPg(1-73) complex. The MNV VPg induced the formation of higher-order multimers or tubular fibrils of RdRp and enhanced the RdRp activity. The replication of MNV mutants with VPg-binding defective RdRps was completely blocked in a cell culture system. Moreover, the crystal structure of the complex provided the evidence that the interaction between VPg and RdRp plays a crucial role in NoV replication through the higher-order multimer formation of RdRp molecules.

## Materials and Methods

### Subcloning, Protein Expression, and Purification

MNV RdRp and different lengths of VPg including VPg(1-73) were cloned into pET14b or pET22b vectors and introduced into *Escherichia coli* ER2566 or BL21 (DE3), as described previously (Han et al., 2010; Lee et al., 2011). VPg(1-124) and eight truncates with different lengths, VPg(1-73, 1-86, 1-96, 1-104, 1-119, 20-124, 40-124, and 64-124), were used to construct clones. Single-amino-acid-changed mutants of R239A, D331A, and L354D of RdRp were constructed via site-directed mutagenesis by PCR amplification (**Table 1**). The purification of MNV RdRp protein used for the structural and biochemical studies has been described previously (Han et al., 2010; Lee et al., 2011). Briefly, protein expression was induced with isopropyl β-D-1-thiogalactopyranoside at 37°C for 4 h or at 15°C overnight. The cells were disrupted by sonication after treatment with DNase and RNase. The recombinant proteins were purified using a nickel-nitrilotriacetic acid (Ni-NTA) affinity and gel filtration chromatography. For the biochemical assay and electron microscopy studies, recombinant RdRp, VPg(1-124) and VPg(1-73) were prepared at 1–1.5 mg/mL. The proteins were quantified by using Nanodrop 1000 (Thermo Scientific) and SDS-PAGE.

**Table 1:** Primers for mutants involved in the RdRp-VPg complex.

Name	Sequence	Purpose
RdRp-F^1^	GGAATTCCATATGCTTCCCCGCCCCTCAGGCACCTAT	RdRp amplification
RdRp-R^1^	ATAAGAATGCGGCCGCATCCTCATTCACγGACTGCTGA	RdRp amplification
RdRp-F^2^	CATATGGGACCCCCCATGCTTCCCC	RdRp amplification
RdRp-R^2^	GGATCCTCACTCATCCTCATTCACγGA	RdRp amplification
R239A-F	CACGCCAATTTCGCATACCACATGGATGCTGAC	Arg^239^ to Ala mutant RdRp
R239A-R	GTCAGCATCCATGTGGTATGCGγTTGGCGTG	Arg^239^ to Ala mutant RdRp
D331A-F	GTAACCCGAGTTGCACCTGACATTGTG	Asp^331^ to Ala mutant RdRp
D331A-R	CACAATGTCAGGTGCAACTCGGGTTAC	Asp^331^ to Ala mutant RdRp
L354D-F	GTTTCGACCAACGAUGAGTTGGATATG	Lys^354^ to Asp mutant RdRp
L354D-R	CATATCCAACTCATCGTTGGTCGγC	Lys^354^ to Asp mutant RdRp
VPg (1-124)-F	GGAATTCCATATGGGγGAAGGGCAAGAACAAGAAG	
VPg (1-124)-R	CCGCTCGAGCTCγGTTGATCTTCTCGCCGTA	
VPg(20-124)-F	GGAATTCCATATGCTCACGGATGAGGAGTACGATGAA	
VPg(20-124)-R	CCGCTCGAGCTCγGTTGATCTTCTCGCCGTA	
VPg (40-124)-F	GGAATTCCATATGTCCATTGATGATTACCTCGCTGAC	
VPg (40-124)-R	CCGCTCGAG CTCγGTTGATCTTCTCGCCGTA	
VPg (64-124)-F	GGAATTCCATATGTTCGGGGATGGCTTCGGGTTGAAG	
VPg (64-124)-R	CCGCTCGAGGCCCAGTTTGGCTCTCTCTGCCTT	
VPg (1-73)-F	GGAATTCCATATGGGγGAAGGGCAAGAACAAGAAG	
VPg (1-73)-R	CCGCTCGAGCCCGAAGCCATCCCCGAAGATAGCCTC	
VPg (1-86)-F	GGAATTCCATATGCTCACGGATGAGGAGTACGATGAAG	
VPg (1-86)-R	CCGCTCGAGGCCCAGTTTGGCTCTCTCTGCCTT	
VPg (1-96)-F	GGAATTCCATATGTCCATTGATGATTACCTCGCTGAC	
VPg (1-96)-R	CCGCTCGAGGCGGGCGCGGATGTCGCCACCAGA	
VPg (1-104)-F	GGAATTCCATATGTTCGGGGATGGCTTCGGGTTGAAG	
VPg (1-104)-R	CCGCTCGAGGGGGCCAACCACATTCCAGTCGAT	
VPg (1-119)-F	GGAATTCCATATGTTCGGGGATGGCTTCGGGTTGAAG	
VPg (1-119)-R	CCGCTCGAGCTCGCCGTAGTCGACCTGGCGGTC	


*^*1,2*^RdRp constructs were cloned into the pET22b vector with C-terminal His-taq^*1*^ or the pET14b vector with N-terminal His-taq^*2*^*.

### Cross-Linking Assays

Inter- and intramolecular interactions between RdRp native or mutants and VPg were tested by glutaraldehyde cross-linking assay. The reaction mixture containing 50 mM HEPES (pH 7.4), 5 mM MgCl_2_, 5 mM DTT, 1 μM oligo(A)_15_, 2 μM RdRp, and 6 μM VPg, was incubated with or without 0.5% SDS. The reaction mixture was treated with 0.001% glutaraldehyde and analyzed by SDS-PAGE followed by western blotting with anti-RdRp or anti-VPg antisera (from immunizing rabbits, Cosmo Genetech, Seoul, South Korea).

### Electron Microscopy

The reaction mixture was placed on carbon-coated copper grids, followed by staining with 0.75% uranyl formate. Images were collected on a 4K × 4K Eagle HS CCD camera (2.1 A/pixel) on a Tecnai T120 microscope (FEI) operating at 120 kV. The defocus and nominal magnification for all images were 1.5 μm and ×52,000, respectively (pixel size: 2.10 Å). In order to identify the RdRp hexamer, a total of 5,149 particles were semi-automatically selected from 150 micrographs using EMAN2 boxer and bad particles were manually excluded (Tang et al., 2007), and it was subjected to two-dimensional (2D) reference-free alignment, multivariate statistical analysis (MSA), and MSA classification, which were iterated by using the IMAGIC software (van Heel et al., 1996). Five representative class average images were compared with the corresponding forward projection images of the hexamer model.

To observe the multimerization, samples were prepared for electron microscopic studies by mixing (a) 2 μM RdRp, 6 μM VPg(1-73) or 1 μM VPg(1-124) and 16 μM oligo(A)_8_, (b) 2 μM RdRp, 6 μM VPg(1-73) or 1 μM VPg(1-124), (c) 2 μM RdRp or 6 μM VPg(1-73) or 1 μM VPg(1-124), and (d) 2 μM RdRp or 6 μM VPg(1-73) or 1 μM VPg(1-124) and 16 μM oligo(A)_8_, in the presence of 2 mM UTP, 2 mM MnCl_2_, and 2 mM MgCl_2_. For mutants, 2 μM of R239A, D331A, or L354D protein was used instead of the native protein. The reaction mixture was incubated for 1 h at room temperature, and then 3 μL of the reaction mixture was placed on carbon-coated copper grids.

### *In Vitro* Binding Affinity Using SPR and RdRp Assay

In order to confirm the binding of VPg to RdRp native or mutants, SPR experiments were carried out to determine the affinity (*K*_D_) of the protein–protein interactions for MNV RdRp-RdRp or RdRp-VPg by using the Biacore2000 system (GE Healthcare, Korea Basic Science Institute, Seoul Branch). RdRp or mutant proteins in sodium acetate buffer (pH 5.0) were immobilized onto a CM5 chip after pH scouting, regeneration scouting, and chip activation with 1-ethyl-3-(3-dimethylaminopropyl)carbodiimide/*N*-hydroxysuccinimide. Proteins were injected onto the chip by the kinetics method after being serially diluted in 20 mM Tris–HCl, pH 8.0, 100 mM NaCl, and 5 mM MgCl_2_. The affinity constant *K*_D_ was determined from the association (*k*_a_) and dissociation (*k*_d_) rates, by evaluation of the 1:1 Langmuir binding model kinetics in the sensorgrams. The RdRp assay was performed as described previously (Han et al., 2010), with minor modifications.

### *In Vitro* RNA-Dependent RNA Polymerase Assay

Template DNAs for the *in vitro* transcription of sgRNA for RNA polymerase assay were amplified by PCR and the RdRp assay was performed as described previously (Han et al., 2010, 2017), with minor modifications. For RdRp assay with (+)sgRNA as a template, 50 μL of reaction mixture containing 50 mM HEPES, pH 7.4, 5 mM MgCl_2_, 10 mM dithiothreitol (DTT), 1 μg of *in vitro* transcribed (+)sgRNA, 250 μM of each NTP, 2.5 μCi [α-^32^P]UTP (3000 Ci mmol^-1^, 10 mCi/mL), and 1 μg RdRp (0.33 μM) was incubated with 0.6–2.4 μg (0.7–2.8 μM) of VPg, or with BSA as a control, for 1 h at 37°C. The reaction was stopped by adding an equal volume of 200 mM EDTA (pH 8.0), and the RNA was extracted by using the RNeasy Mini Kit and analyzed by agarose gel electrophoresis in Tris–borate–EDTA buffer. After electrophoresis, the gel was dried and the [α-^32^P]UTP labeled RNA was detected with a BAS-1500 imaging system (Fujifilm, Tokyo, Japan).

### Crystallization and Structure Determination

MNV RdRp protein was incubated with VPg at a molar ratio of 1:2 in the presence of 2 mM uridine triphosphate (UTP) or guanidine triphosphate (GPT), 10 nM oligo(A) (A_8∼10_) or oligo C (C_6∼10_), 2 mM MnCl_2_, and 2 mM MgCl_2_ at 4°C overnight. The hanging drop vapor-diffusion method was used for the crystallization by mixing complex protein and 0.1 M cacodylate (pH 6.5) buffer containing 1.0 M sodium citrate. Triangular-shaped crystals in the P2_1_ space group appeared within a week, with six monomers in the asymmetric unit. Diffraction data were collected using synchrotron radiation sources at beamline 17A of Photon Factory (Tsukuba) and 5C of PAL (Pohang, South Korea), with the crystals flash-cooled at 100 K in a stream of liquid N_2_.

The data were integrated, scaled, and processed using the HKL2000 program (Otwinowski and Minor, 1997). The initial model of the complex structures was built using molecular replacement and AutoBuild from the PHENIX suite (Adams et al., 2010), employing a previously solved native structure (PDB 3QID) as a search model. Initial difference Fourier maps of the RdRp-VPg(1-73)-RNA complex, with coefficients 2| F_o_| -| F_c_| and | F_o_| -| F_c_|, showed an elongated extra density at the interface of the RdRp hexamers that could be interpreted as VPg, mainly in α-helical conformation. VPg was built manually in the Coot program (Emsley and Cowtan, 2004). Using a high-resolution structure as a template, exploiting geometric redundancies (NCS) and B-sharpening (Brunger et al., 2009) did not improve the electron density maps sufficiently to build a complete VPg model. The VPg structure was verified by examining a composite simulated annealing omit map at the 1.2σ level. The *R* and *R*_free_ values for RdRp-VPg(1-73)-RNA were 20.3 and 24.1%, respectively, after rigid body, NCS, TLS refinements coupled with Ramachandran restraints using the PHENIX program (Brunger et al., 2009; Adams et al., 2010). Ramachandran analysis revealed 97.9, 1.9, and 0.2% in the favored, allowed, and outlier regions, respectively. The structural figures were generated with PyMOL.^1^ The data quality and refinement statistics are presented in **Table 2**.

### Production of Plasmid-Derived Recombinant MNV

The expression cassette of MNV CW3 strain was subcloned from pCW3 (Strong et al., 2012) to pcDNA3 via restriction enzyme cloning. This pcDNA3-MNV system was used to produce recombinant MNV with mutated RdRp. Specific RdRp mutations were generated using QuikChange II Site-Directed Mutagenesis kit (Agilent Technologies) according to the manufacturer’s instructions; the sequence of wild type RdRp were modified to R239A (from TTCAGGTAC to TTCgcaTAC), D331A (from GTTGACCCT to GTTGcaCCT), L354D (from AACCTCGAG to AACgatGAG), and DDAA (from GGTGATGACGAG to GGTgcggcgGAG). Five hundred nanograms of the recombinant plasmids were transfected into 293T-CD300LF cells (1 × 10^5^ cells/well in 24-well plate) using Lipofectamine 2000 (ThermoFisher Scientific), and the transfected cells were harvested by freezing at 24, 48, and 72 h post-transfection (hpt) to titrate produced infectious viruses. Four micrograms of the recombinant plasmids were transfected into 293T-CD300LF cells (5 × 10^5^ cells/well in 6-well plate) using standard Calcium/Phosphate transfection method, and the transfected cells were lysed to analyze expressed viral proteins; were lysed in TRI reagent (Sigma-Aldrich, T9424) at 24, 48, and 72 hpt to measure viral genome replication and transcription; and fixed on coverslips with PBS containing 2% formaldehyde (Ted Pella) at 24, 48, and 72 hpt to examine the localization of viral proteins and dsRNAs.

### Cell Culture

293T cells transduced with the MNV receptor CD300LF (293T-CD300LF) (Orchard et al., 2016) were used to analyze MNV replication and viral gene expression upon transfection of plasmids expressing recombinant MNVs. BV-2 cells were used to titer infectious virus for TCID_50_ analysis. Both cell lines were cultured as previously described (Hwang et al., 2012).

### Western Blot Analysis

The transfected cells were lysed with sample buffer (0.1 M Tris, pH 6.8, 4% SDS, 4 mM EDTA, 286 mM 2-mercaptoethanol, 3.2 M glycerol, and 0.05% bromophenol blue). The resultant cell lysates were resolved by SDS-PAGE and proteins were specifically detected using the following antibodies: rabbit anti-ProPol [detecting both protease and polymerase (RdRp) of MNV] (Hwang et al., 2012) and anti-Actin.

### Tissue Culture Infectious Dose 50 (TCID_50_)

The transfected cells with media were harvested by freezing and lysed by one cycle of freeze-and-thaw. The infectious viruses were tittered via TCID_50_ analysis as previously described (Biering et al., 2017).

### Quantitative PCR

The transfected cells were lysed in TRI reagent and RNAs were extracted and column purified (Zymo Research) after DNAse treatment according to the manufacturer’s instruction. cDNAs were reverse transcribed from 1 μg of the DNAse-treated RNAs using IMPROM-II reverse transcriptase (Promega) according to the manufacturer’s instruction. Quantitative PCR analysis was conducted as previously described (Biering et al., 2017) using SYBR-green reagents with the following sets of primers: 5′-AGCTCAGGATGGTCTCGGAT-3′ and 5′-TCAAGAGCAAGGTCGAAGGG-3′ for both positive and negative-strand of MNV, 5′-GAGTCAACGGATTTGGTCGT-3′ and 5′-TTGATTTTGGAGGGATCTCG-3′ for GAPDH.

### Immunofluorescence Analysis

The transfected cells were fixed on coverslips and permeabilized with PBS containing 0.05% Saponin and blocked and probed in PBS containing 0.05% Saponin and 5% normal donkey serum, which were mounted on glass slides with ProLong Diamond Antifade Mountant (Life Technologies). Nuclei were stained with Hoechst 33342 and the following antibodies were used: guinea pig anti-ProPol (Hwang et al., 2012), rabbit anti-VPg, rabbit anti-VP1 (Hwang et al., 2012), mouse anti-dsRNA, Alexa Fluor 647 Donkey anti-Guinea Pig, Alexa Fluor 488 Donkey anti-Rabbit, and Alexa Fluor 555 Goat polyclonal anti-Mouse. Images were acquired using the EVOS FL Cell Imaging System.

## Results

### Chracterization of Higher-Order Structures of RdRp Mediated by VPg

Our initial attempt to prepare the MNV proteins of RdRp and VPg was not successful due to the instability of the full-length VPg(1-124) and RdRp. The N- and C-terminal regions of VPg were predicted to be disordered according to the disorder prediction program (Prilusky et al., 2005), similar to the VPg of rabbit hemorrhagic disease virus and Sapporo virus in the *Caliciviridae* family (Leen et al., 2013). Nine constructs with various deletions at the N- or C-termini of the full length VPg(1-124) were prepared (**Table 1**). Among them, VPg(1-73) was found to be stable (Supplementary Figure S1). RdRp tends to readily form aggregates at high concentrations and the experiments were performed at the concentration where it is readily soluble.

**FIGURE 1:**
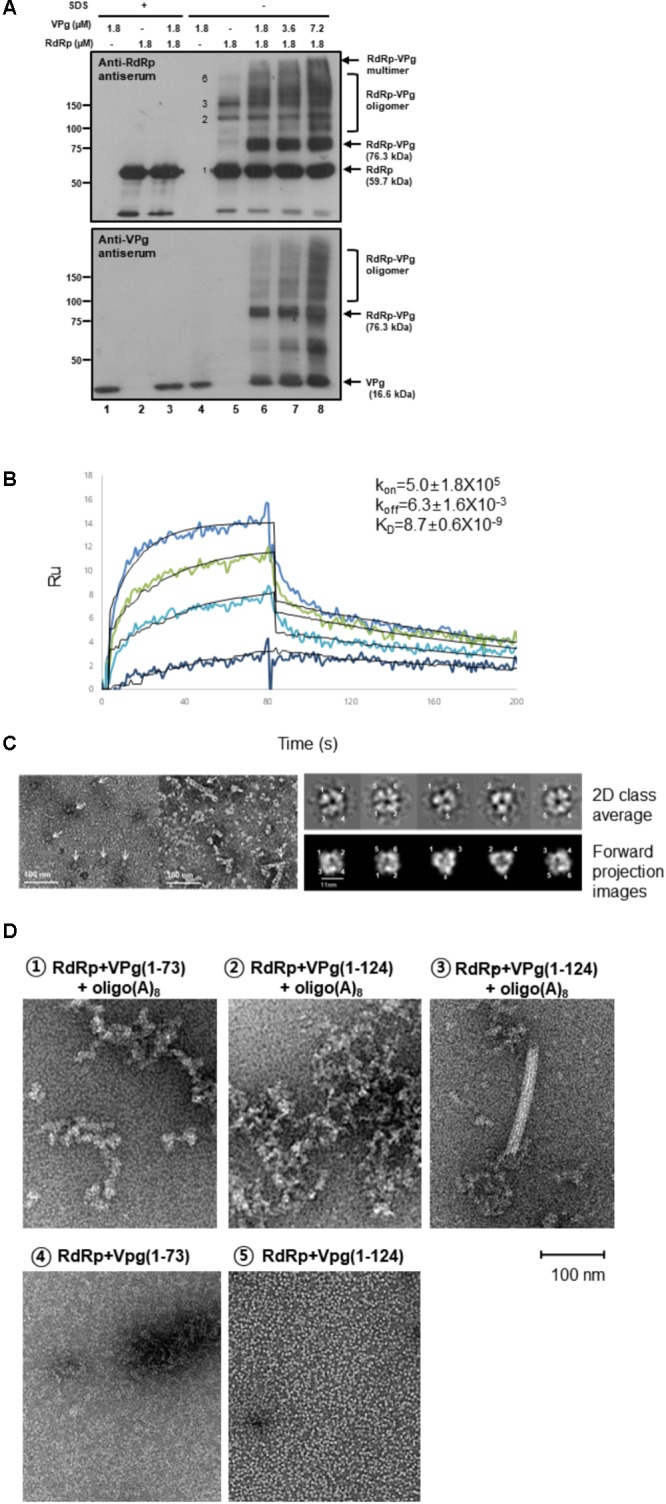
Interactions in the RdRp-VPg(1-73) complex. **(A)** Cross-linking studies of RdRp-VPg-RNA complexes. The mixture containing 50 mM HEPES (pH 7.4), 5 mM MgCl_2_, 5 mM DTT, 1 μM oligo(A)_15_, 2 μM RdRp, and 6 μM VPg was incubated with or without 0.5% SDS. The reaction mixture was cross-linked by 0.001% glutaraldehyde treatment at room temperature for 6 min. Proteins were separated on 8% SDS-PAGE and analyzed by western blotting with anti-RdRp or anti-VPg antisera. The molecular masses of standard proteins are shown in kDa unit on the left. The RdRp, VPg monomer, and RdRp-VPg complex bands are indicated on the right of the gel. Monomer, dimer, trimer, and hexamer bands of RdRp were indicated with numbers 1, 2, 3, and 6, respectively. The experiments were repeated in triplicates. **(B)** SPR binding assay. RdRp or mutant proteins in sodium acetate buffer (pH 5.0) were immobilized onto a CM5 chip after being serially diluted in 20 mM Tris–HCl, pH 8.0, 100 mM NaCl, and 5 mM MgCl_2_. The binding affinity constant (*K*_D_) was determined from the association (*k*_on_) and dissociation (*k*_off_) rates by evaluating the 1:1 Langmuir binding model kinetics in the sensorgrams. Colored curves depict experimental data at different analyte concentrations and fitted curves modeled to describe a 1:1 binding event are overlaid in black. The experiments were duplicated starting from two separate protein preparations and each measurement performed in triplicate. **(C)** Electron microscopic studies of RdRp-VPg-RNA complexes. (Left panel) Hexamers of MNV RdRp are shown on the left (pointed by arrows) and hexameric oligomers are shown on the right (arrows). 1–6 μM proteins were used and the reaction mixture was incubated for 1 h at room temperature, and the reaction mixture was placed on carbon-coated copper grids. Images were collected with a 4K × 4K Eagle HS CCD camera on a Tecnai T120 microscope (FEI, Eindhoven, Netherlands) operating at 120 kV. The scale bar represents 100 nm. (Right panel) Single particles of RdRp hexamers are represented as 2D class averages and (lower panel) corresponding forward projection images. The six monomers of RdRp are numbered from 1 to 6 based on crystal structures in **Figure 2D**. **(D)** Electron microscopic images of the RdRp-VPg(1-73)-oligo(A)_8_ or RdRp-VPg(1-124)-oligo(A)_8_ complexes are shown (1D, 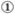 and 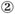). An RdRp tubular fibril (approximately 18 nm in diameter) formed after incubation with VPg(1-124) and oligo(A)_8_ is shown (1D, 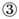). A comparison between VPg(1-73) and VPg(1-124) in complex with RdRp is shown in the absence of oligo(A)_8_ (1D, 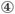 and 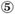).

We initially examined the RdRp–VPg interaction using chemical crosslinking with glutaraldehyde, sodium dodecyl sulfate polyacrylamide gel electrophoresis (SDS-PAGE) and immunoblotting. RdRp was found to form dimers (119.4 kDa), trimers (179.1 kDa), and hexamers (358.2 kDa) (**Figure 1A**, lane 5), whereas VPg existed as a monomer (lane 4). Protein bands of approximately 76 kDa were detected by both anti-RdRp and anti-VPg antisera when VPg was incubated with RdRp (lanes 6–8), matching the sum of the RdRp (59.7 kDa) and VPg (16.6 kDa) masses. This 76 kDa band was not detected when RdRp or VPg was incubated separately (lanes 4 and 5) or when the protein interaction was interrupted by the addition of 0.5% SDS (lanes 1–3). Protein bands with molecular masses higher than the RdRp hexamer were observed upon addition of VPg in a dose-dependent manner (lanes 6–8).

To quantitatively examine the interaction between RdRp and VPg, surface plasmon resonance (SPR) assays were carried out to measure binding affinities using immobilized RdRp. The affinity constants (*K*_D_) were 2.2 ± 1.1 and 8.7 ± 0.6 nM for the RdRp–RdRp and RdRp–VPg(1-124) interactions, respectively (data not shown and **Figure 1B**), indicating a comparable binding affinity between RdRp and VPg(1-124) to that between RdRp molecules. The *K*_D_ for RdRp-VPg(1-73) complex was 17 ± 0.4 nM (data not shown), which is slightly lower affinity than that of the RdRp-VPg(1-124) complex, suggesting a role of the disordered C-termini of VPg in its interaction with RdRp.

The interaction of RdRp with VPg was further examined using transmission electron microscopy. The RdRp molecules by themselves were found to form densely packed individual hexamers or fibrous hexameric oligomers like hexamers in tandem stacks (**Figure 1C**, left panel, arrows). The formation of hexamers was supported by 2D averaged images (**Figure 1C**, right panel). Interestingly, addition of VPg(1-73) or VPg(1-124) induced the formation of aggregates or higher-ordered structures of RdRp in the presence of oligo(A)_8_ RNA, although less with VPg(1-73) than with VPg(1-124) (**Figure 1D**). No aggregate was observed with either VPg or RdRp alone (data not shown), although in the presence of oligo(A)_8_ the aggregated forms of RdRp or VPg were occasionally observed (**Figure 1D**, lower panel). Notably, RdRp often formed a fibril-like structure in the presence of VPg(1-124) and oligo(A)_8_ (**Figure 1D**, upper right). Such fibril formation of RdRp was also observed with the RdRp of FMDV and poliovirus (Spagnolo et al., 2010; Lee et al., 2011; Bentham et al., 2012).

### Crystal Structure of the RdRp-VPg(1-73)-RNA Complex

**FIGURE 2:**
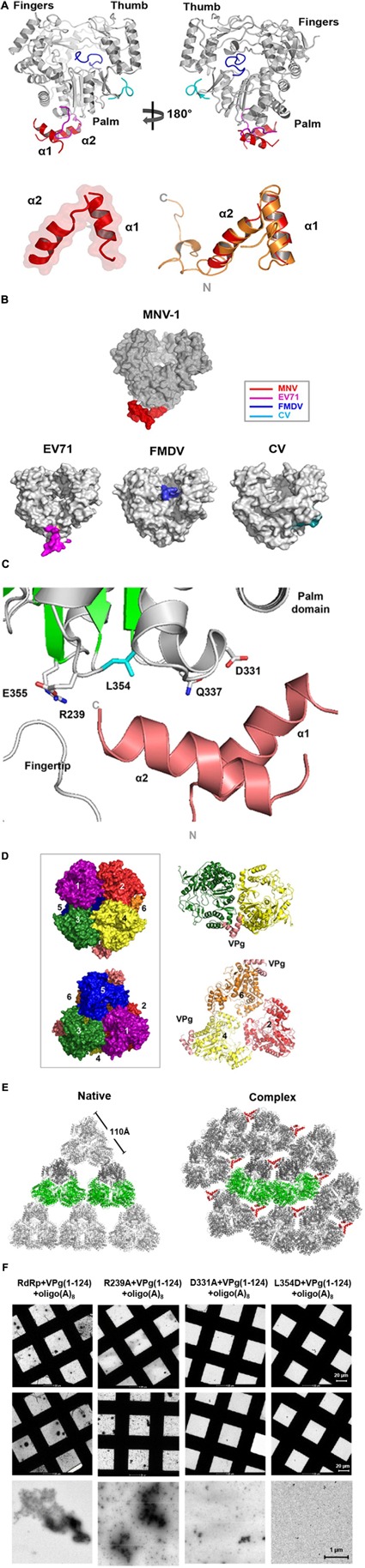
Structures of the MNV RdRp-VPg complex. **(A)** The RdRp-VPg(1-73) complex is shown in gray (RdRp) and red (VPg) (upper panel). Helical structure of VPg is shown below, where it was superimposed with the NMR structure (orange). **(B)** VPg is bound at the bottom of the palm domain of RdRp. Surface representations of VPg structures from MNV, EV71, FMDV, and CV are shown with red, pink, blue, and cyan color, respectively, highlighting diverse VPg binding modes (lower panel). **(C)** The interaction of RdRp with VPg in the RdRp-VPg(1-73) complex in which VPg binds to the base of the palm domain of RdRp. The critical amino acid residues (e.g., Asp331, Gln337, Leu354, and Glu355) of RdRp involved in the interaction are shown. **(D)** Surface representation of the hexameric RdRp-VPg complex is shown in the box, where a side view along the two-fold axis (upper) and a top view along the threefold axis (lower) are shown. Six monomers of RdRp are numbered from 1 to 6 and colored in rainbow colors, whereas VPg is in pink tint. The dimeric (upper panel) and trimeric (lower panel) arrangements found in the RdRp hexamer complex are shown in ribbon diagram on the right. **(E)** The crystal packing of the structures of RdRp alone (PDB ID 3QID) (left panel) and the RdRp-VPg(1-73) complex (right panel) are shown at the threefold axis. **(F)** Electron microscopic images of the RdRp-VPg(1-124)-oligo(A)_8_ complexes using the native, R239A, D331A, and L354D RdRps (from the left). The conditions of protein concentrations and procedures are the same as in **Figures 1C,D**. Scale bars are noted.

Next, we determined the complex structure of RdRp-VPg(1-73) at 3.1 Å resolution (**Table 2**). Continuous electron density was observed at the base of the palm domain of RdRp in the initial map. Two helix backbones (α1 and α2) modeled for VPg were fitted to the electron density with lower B factor (**Figure 2A**), which was verified by examining a simulated annealing composite omit map at the 1.2σ level (Supplementary Figure S2). However, the electron densities for the side chains of VPg were not well defined, whereas those of RdRp were clearly interpretable. Superposition of the VPg backbone in the complex with previously determined nuclear magnetic resonance (NMR) structure of MNV VPg(11-85) (PDB 2M4G) gave the root-mean-square deviation (RMSD) of 1.5 Å based on secondary structure matching (Leen et al., 2013), indicating little change in VPg structure upon the complex formation. MNV VPg had a distinct helix-loop-helix conformation and bound to the palm domain of RdRp, and the binding site was relatively similar to that of EV71 RdRp (Chen et al., 2013) but far from that of VPg at the active site of FMDV or the thumb domain of CV RdRps (**Figure 2B**) (Ferrer-Orta et al., 2006; Gruez et al., 2008). Viral VPgs show diverse conformations among the members of the same family (Ferrer-Orta et al., 2006; Gruez et al., 2008; Chen et al., 2013); the VPg proteins of EV71, FMDV, and CV in the *Picornaviridae* family exhibit V-shaped, loop-rich, and extended conformations, respectively. In contrast, viral RdRps have very similar structure; superposition of the RdRp structures of MNV, EV71, FMDV, and CV gave RMSD values of 2.5–2.7 Å. At the interface between VPg and the palm domain of RdRp, the side chains of MNV RdRp were well-resolved (**Figure 2C**). Although the sequences of RdRp residues at the interface with VPg were not highly conserved in the members of the *Caliciviridae* and *Picornaviridae* (Supplementary Figure S3), Asp331 and Leu354 were selected for a mutational analysis to examine their roles in RdRp–VPg interactions. The electron density for the oligo(A)_8_ RNA was not visible in the complex structure, despite the presence of the oligonucleotide in the crystallization solution. Superposition of the complex with the HuNV RdRp-RNA complex (3BSO) suggested a potential location of RNA at the active site and within the closed conformation of fingers and thumb domains (Zamyatkin et al., 2008; Han et al., 2017).

**Table 2:** Data collection and refinement statistics.

	RdRp-VPg(1-73)-RNA complex
**Data statistics**	
Space group	P2_1_
Cell dimensions	
*a*, *b*, *c* (Å)	109.3, 159.8, 121.7
*α*, *β*, *γ* (°)	90.0, 97.2, 90.0
Resolution (Å)	50-3.1
*R*_sym_ or *R*_merge_	19.8 (51.9)^a^
*I*/σ	5.2 (1.4)^a^
Completeness (%)	96.4 (90)^a^
Redundancy	3.6 (2.4)^a^
**Refinement statistics**	
Resolution (Å)	3.1
No. of reflections	69,633
*R*_work_/*R*_free_	20.3/24.1
No. of atoms	23,161
RdRp	22,819
VPg	324
Water	18
*B*-factors^b^	
RdRp	54.5
VPg	65.8
Water	29
Root-mean-square deviations	
Bond lengths (Å)	0.004
Bond angles (°)	0.545


^*a*^*Values in parentheses are for the highest-resolution shell. RdRp-VPg(1-73)-RNA: RdRp complexed to VPg(1-73) in the presence of RNA. ^*b*^Average B value for whole chain atoms*.

### Spatial Arrangement of the RdRp-VPg(1-73) Complex

The overall structure of MNV RdRp in the RdRp-VPg(1-73) complex was very similar to that in the native RdRp and RdRp-ligand complex structures as previously reported (Lee et al., 2011; Alam et al., 2012). A large ball-like hexameric arrangement of RdRp was observed in the crystal structures (**Figure 2D**). A face-to-face contact of the palm domains was capable of forming a dimeric structure, and the trimer interface was largely made up of the thumb and fingers domains in the hexameric complex. Previous size-exclusion chromatography and cross-linking assays showed that MNV RdRp exists primarily as a monomer in room temperature solution but oligomerize at 37°C, resulting in heavy precipitation (Lee et al., 2011; Alam et al., 2012). HuNV RdRp showed dimeric arrangements only in its crystal structure (Hogbom et al., 2009).

The RdRp-VPg(1-73) complex structure showed that the RdRp hexamers were more closely packed in the presence of the VPg(1-73). When viewed from the top along the threefold axis, the hexamers in the native were aligned to form a triangular shape via interactions at the triangle vertices (**Figure 2E**, left). In contrast, those in the RdRp-VPg(1-73) complex were packed more tightly, face to face, by six neighboring hexamers, which could drastically alter the RdRp hexamer clustering (**Figure 2E**, right). Further, the interactions between RdRp molecules in the RdRp-VPg(1-73) complex were distinct from that of the native; interactions between Arg329 and Gln389 and between Asp91 and Arg411 of adjacent RdRp molecules were observed in the RdRp-VPg(1-73) complex structure but absent in the native (Supplementary Figure S4).

D331A and L354D mutants did not promote the formation of higher-order structures of RdRp in the presence of VPg and oligo(A)_8_, suggesting that the point mutations inhibited the interaction of RdRp with VPg and consequently the multimerization process (**Figure 2F**). An R239A mutant of RdRp was prepared as a control for D331A and L354D mutants, which is located near the interface but not involved in the interaction between RdRp and VPg in the complex model (**Figure 2C**), and it induced multimerization as significantly as the native. When we compared the binding of D331A and L354D mutants to VPg(1-124) with that of the WT RdRp by a Langmuir 1:1 binding kinetic analysis, the binding affinity of the mutants was significantly decreased to 23 ± 0.8 and 210 ± 1.6 nM, respectively, but not completely abolished (Supplementary Figure S5). Therefore, these results strongly suggested that the higher-order multimerization or tubular fibril formation of RdRp molecules was mediated by VPg and that Asp331 and Leu354 of RdRp were involved in the interaction of RdRp with VPg for the multimer formation in the presence of RNA.

Since the exact residues of VPg at the interface were not discernible in the electron density maps due to low resolution, the side chains from the known VPg structure (PDB 2M4G) were used to deduce the residues of VPg at the interface. Accordingly, L55D of VPg was prepared as a potential non-interactor of RdRp and examined for its effect on the multimerization of RdRp. However, we did not observe any difference between the native VPg and VPg/L55D mutant via cross-linking assays (data not shown), indicating the necessity of a better structural resolution of VPg for its functional study in RdRp multimerization.

### RdRp–VPg Interaction in RNA Synthesis and Viral Replication

**FIGURE 3:**
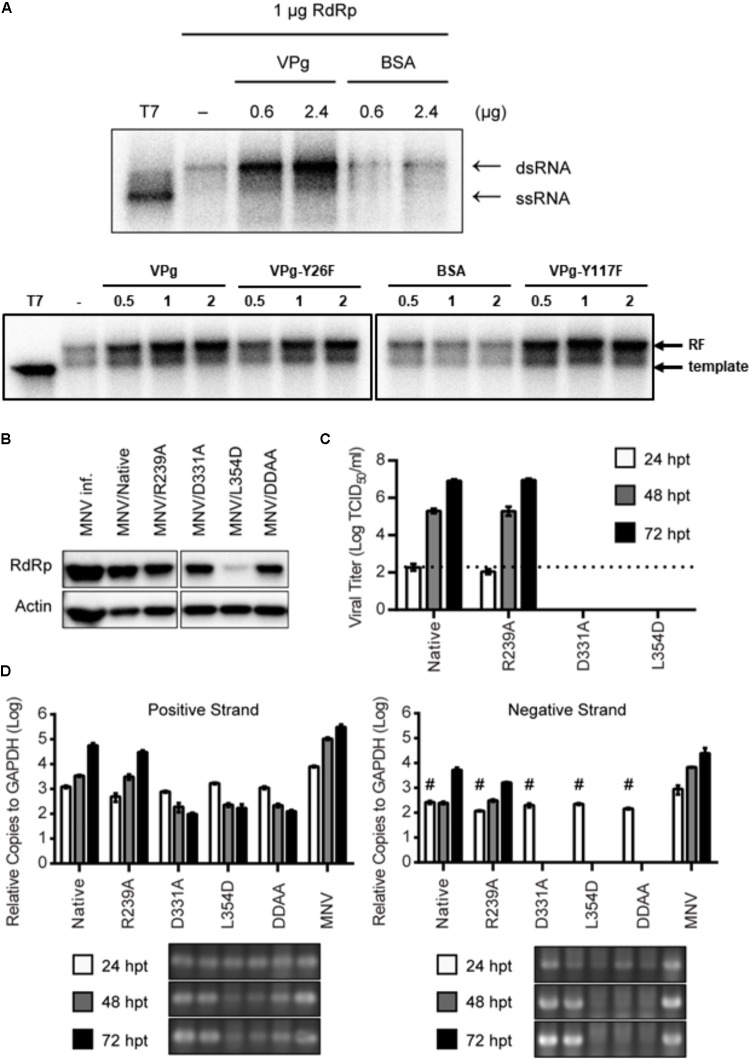
RdRp–VPg interaction for RNA synthesis and viral replication. **(A)** Representative autoradiograph of *in vitro* RNA synthesis assay. The RNA synthesis was monitored by the generation of double strand RNA (dsRNA) from poly(A) tailed sgRNA template in the presence of [α-^32^P]UTP. T7 indicates T7 RNA polymerase as a non-polymerizing control (upper panel). The experiments were repeated in duplicates. The RNA polymerase activity of recombinant RdRp (1 μg) in 50 mM HEPES, pH 7.4, 5 mM MgCl_2_, 10 mM dithiothreitol (DTT), 1 μg of *in vitro* transcribed (+)sgRNA, 250 μM of each NTP, 2.5 μCi [α-^32^P]UTP was determined in the presence of VPg native, Y26F or Y117F, analyzed by agarose gel electrophoresis in Tris–borate–EDTA buffer (lower panel). BSA was used as a control. **(B)** Representative western blot data showing the expression of RdRp at 24 hpt of plasmids producing the indicated recombinant MNVs into 293T-CD300LF cells. As a positive control, the cells were infected with MNV at the multiplicity of infection (MOI) of five for 24 h (MNV inf.). Actin as loading controls. *N* = 2 replicates. **(C)** Growth analysis of recombinant MNVs upon transfection of 293T-CD300LF cells with plasmids producing MNVs with the indicated mutations. Data as mean ± SEM. Dashed line indicates the limit of detection. *N* = 3 replicates. **(D)** Quantitative PCR analysis for the positive and negative strands of MNV generated at 24, 48, and 72 hpt as described in **(C)**. # in negative strand indicates inclusion of false-positive signals due to remaining plasmid DNAs even after DNAse treatment. Gel pictures under bar graph show PCR products after 40 cycles of amplification. As a positive control, the cells were infected with MNV at MOI = 0.05 for 72 h (MNV inf.). *N* = 3 replicates.

We investigated the functional consequence of the VPg-mediated multimerization of RdRp. In an *in vitro* RNA synthesis assay, MNV RdRp synthesized negative-stranded RNA *de novo* from poly(A) tailed subgenomic RNA (sgRNA), consequently forming double-stranded RNA (**Figure 3A**). Upon addition of purified VPg into the reaction mixture, the formation of double-stranded RNA was substantially increased in a dose-dependent manner. In contrast, the addition of an unrelated protein (bovine serum albumin, BSA) into the reaction mixture did not affect the RNA synthesis. To determine the role of VPg priming in this VPg-mediated enhancement of RdRp activity, priming-defect mutants of VPg were subjected to the same RNA synthesis assay. Both Y26F and Y117F mutants blocking the canonical and non-canonical guanylylation (Han et al., 2010; Subba-Reddy et al., 2011), respectively, enhanced the *in vitro* RNA synthesis like the wild type VPg, indicating that the VPg-mediated enhancement of RNA synthesis was independent of VPg-priming (**Figure 3A**, lower panel).

To examine the role of the RdRp–VPg interaction in the lifecycle of MNV, we utilized a plasmid-based reverse-genetic system of MNV (Strong et al., 2012); we mutated the relevant sequences in the plasmid to generate recombinant MNVs harboring R239A, D331A, or L354D mutants of RdRp. We also made an MNV plasmid with D346A/D347A (henceforth DDAA) mutations in RdRp as a non-replicating control; DDAA is a polymerase-activity-dead mutant of RdRp (Lee et al., 2011). We transfected these recombinant plasmids into 293T cells stably expressing the MNV receptor CD300LF (293T-CD300LF) (Orchard et al., 2016) for efficient transfection and amplification of produced virus. In the transfected cells, all RdRp mutants were properly processed and expressed like native RdRp in the MNV-infected cells, although there was less RdRp/L354D compared to the others (**Figure 3B**). Strikingly, we were not able to recover any infectious MNV from the plasmids containing D331A or L354D mutants of RdRp (**Figure 3C**). In contrast, the recombinant MNV with the RdRp/R239A mutation replicated similarly to the control plasmid-derived MNV with native RdRp. We further checked the synthesis of positive and negative strand MNV RNA from the transfected plasmids (**Figure 3D**). Positive strand MNV RNA was detected from all constructs including the polymerase-dead mutant (DDAA), indicating a basal-level signal of positive strand MNV RNA from remaining plasmid DNAs and the active CMV promoter of all constructs. However, the plasmids with the native or R239A RdRp produced about 1,000-fold more positive strand RNA at 72 hpt. The data suggested that there was an approximately 1,000-fold amplification of the MNV genome by RdRp only from the plasmid-derived MNV with the native or R239A mutant RdRp. Consistently, we detected the specific synthesis of negative strand MNV RNA, the intermediate of the MNV genome replication, only in the cells transfected with MNV plasmids with the native or R239A mutant of RdRp (**Figure 3D**). Taken together, these data suggested that the RdRp–VPg interaction was required for MNV replication in the host cell.

**FIGURE 4:**
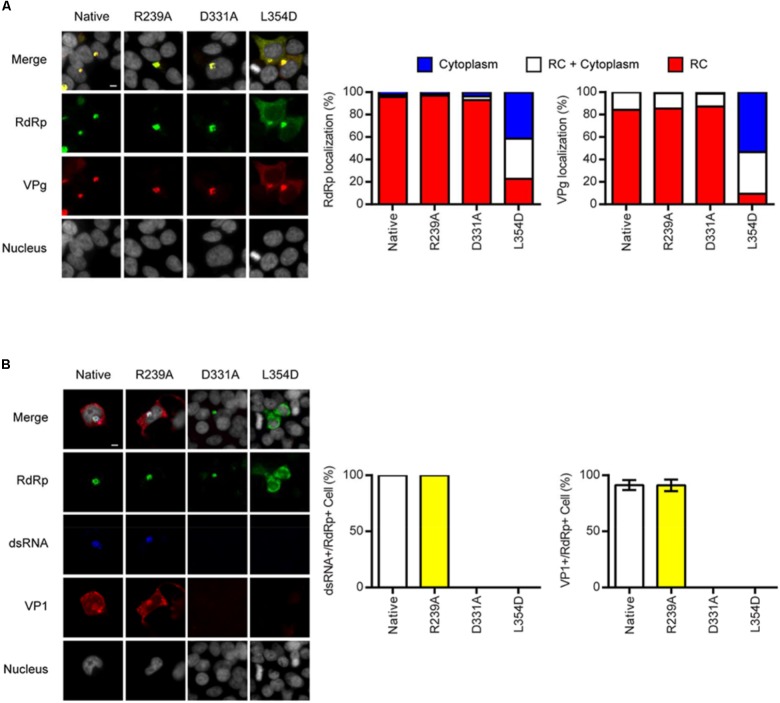
Essential role of RdRp–VPg interaction in viral replication. **(A)** Immunofluorescence assay for the localization of RdRp and VPg at 24 hpt of 293T-CD300LF cells with plasmids producing MNVs with the indicated mutations. Representative images (left) and quantitation (middle and right). Data as mean. *N* = 3 replicates. **(B)** Immunofluorescence assay for the localization of RdRp, dsRNA, and VP1 at 72 hpt as described in **(A)**. Representative images (left) and quantitation (middle and right). Data as mean ± SEM. *N* = 2 replicates.

To understand the essential function of the RdRp–VPg interaction, we examined the steps of MNV replication in the cell. MNV non-structural proteins generated from the proteolytic processing of ORF1 (Sosnovtsev et al., 2006) reorganize the cellular membranes to form replication compartment (RC), where RdRp replicates the MNV genome (Wobus et al., 2004); at 24 hpt, RdRps from all the plasmid-derived MNVs showed perinuclear localization, a typical pattern of MNV RC (Hwang et al., 2012) (**Figure 4A**). RdRp/L354D showed substantial cytoplasmic localization compared to the other RdRps, suggesting that the L354D mutation of RdRp might interfere with forming and/or maintaining the RC structure. VPgs from the plasmid-derived MNVs showed localization patterns similar to those of corresponding RdRps. Collectively, these data suggested that the RdRp–VPg interaction was not required to form and/or maintain the MNV RC *per se*, although the L354 residue of RdRp might play an additional role in the RC structure. In all the transfected cells, RdRps were still detectable and showed the 24 hpt-like localization pattern at 72 hpt (**Figure 4B**). However, double-strand RNA (dsRNA), the intermediate of +RNA virus replication, was detected only at the RCs of plasmid-derived MNV with the native or R239A mutant RdRp, even though only a few cells survived the cytopathic effect of MNV replication; VP1, the structural protein expressed from the sgRNA of MNV after genome replication (Thorne and Goodfellow, 2014), showed the same expression pattern and was detectable only from MNV with the native or the R239A mutant RdRp. Taken together, our data demonstrated that the RdRp–VPg interaction enhanced the activity of MNV RdRp and was required for the efficient replication of MNV in cells. These data further suggest that the VPg-mediated multimerization of RdRp might be essential for efficient MNV infection in the host.

## Discussion

+RNA viruses can synthesize viral RNAs *de novo* and/or with primers. Caliciviruses utilize both *de novo* and primer-dependent RNA synthesis, in which VPg functions as the proteinaceous primer (Paul et al., 1998; Rohayem et al., 2006). In fact, VPg is a multifunctional protein involved in protein synthesis and viral genome packaging into virions; degradation or mutation of VPg abolishes the infectivity of viral genomic RNA (Burroughs and Brown, 1978; Subba-Reddy et al., 2011). To understand the interaction between this multifunctional VPg and viral RNA polymerase in the lifecycle of MNV, we characterized the interaction of RdRp with VPg(1-73) in the presence of RNA. Higher-order multimers or tubular fibrils of RdRp molecules emerged in the presence of VPg. Moreover, VPg enhanced the polymerizing activity of RdRp and the RdRp–VPg interaction was required for efficient replication of MNV in a cell culture system. MNV RdRp is predominantly a monomer in solution and tends to aggregate slowly when the concentration is higher than 5–6 mg/mL at room temperature. A temperature jump to 37°C results in heavy precipitation of RdRp. On the other hand, when RdRp and VPg are mixed together in the presence of oligo A, rapid aggregation is observed. Like the native RdRp, the D331A and L354D mutants can oligomerize as a hexamer by themselves in the absence of VPg. However, they do not promote the formation of higher-order multimers in the presence of VPg and oligo A. Aggregation of RdRp may follow a slow process of precipitation when the condition is changed, e.g., temperature jump, whereas VPg interaction with RdRp induces the rapid formation of higher-ordered multimers in the presence of RNA. Collectively, our data suggest that the multifunctional VPg might play a crucial architectural role in the higher-order multimerization of RdRp molecules and consequently in MNV replication inside the host.

Here we propose a model of the VPg-mediated RdRp multimerization in MNV. A large ball-like RdRp hexamer is generated through dimeric and trimeric interactions among RdRp molecules. VPg augments the interaction between the RdRp hexamers, which can be closely packed into higher-order multimers or tubular fibrils in the presence of RNA. The construction of RdRp tubular fibrils with a uniform diameter (∼18 nm) leads to the spatial clustering of replication components. The ball-like hexameric RdRp can provide a large inner space where single-stranded RNA can be sequestered. Both the RNA and VPg are required for higher ordered multimer formation of RdRp, which strongly enhances cooperative RNA binding as well as highly efficient replication. RdRp has a high tendency to readily form aggregates at high concentrations (∼100 μM). In contrast, the concentration of RdRp upon multimeriztion by VPg was much lower (∼2 μM).

The crystal structure of the MNV RdRp-VPg(1-73) complex showed a more tightly packed arrangement of RdRp molecules than that of RdRp molecules themselves; a hexamer of RdRp molecules was surrounded compactly by six neighboring hexamers and this was mediated by specific binding of VPg(1-73). Considering the higher affinity of VPg(1-124) than that of VPg(1-73) to RdRp (8.9 nM vs. 17 nM), RdRp molecules might be even more compactly multimerized in the presence of VPg(1-124), thereby leading to different higher-order structures. In fact, the binding of VPg(1-124) to RdRp resulted in a higher degree of multimerization of RdRp than the VPg(1-73), suggesting a potential role of the C-terminal region of VPg in RdRp binding. However, the affinity difference between the full length and the truncated VPgs might be due to simple conformational change of VPg after substantial deletion of the protein, and the C-terminal region of VPg may not play any role in the RdRp–VPg interaction. It is noteworthy that the C-terminal region of VPg is involved in the recruitment of translation initiation protein, eIF4G (Chung et al., 2014). In this regard, the C-terminal region of VPg may hinder the formation of compact structure of RdRp-VPg multimers by bringing other cellular proteins *in vivo*. Alternatively, such interaction between the C-terminal region of VPg with cellular translational machinery may happen only after VPg is linked to viral genome, so it may not affect the formation of RdRp-VPg multimers during replication. Collectively, although we cannot exclude the possibility that the VPg(1-124) might have an alternative way of binding to RdRp, we speculate that the disordered C-terminal region of VPg might further strengthen the interaction of VPg with the large ball-like RdRp hexamers.

The replication of +RNA viruses was proposed to be facilitated by higher-order structure formation of viral RdRps that are immobilized on membranous structures (Spagnolo et al., 2010; Lee et al., 2011; Bentham et al., 2012). Such facilitation might be mediated through augmented avidity of participating polymerases as a group for their substrate RNAs and/or retention of intermediate products for sequential reactions. The formation of planar or tubular oligomeric arrays of RdRp molecules correlate with cooperative RNA binding and elongation and a high efficiency of viral replication (Burroughs and Brown, 1978; Spagnolo et al., 2010; Subba-Reddy et al., 2011; Strong et al., 2012). Likewise, the VPg-mediated formation of tubular fibrils or higher-order multimers of MNV RdRp molecules may increase the clustering of replication components and consequently enhance the replication of MNV. Interestingly, the recombinant MNV with the VPg-binding defective RdRp mutants, D331A or L354D, showed no sign of productive replication in a cell culture system. These data suggest that the defective VPg-binding ability of these RdRp mutants led to impaired multimerization of RdRps and consequently faulty replication of MNV, indicating the necessity of the VPg-mediated higher-order multimerization of RdRp for MNV replication in cells. Considering the decreased yet still substantial binding affinities of the D331A and L354D RdRp mutants to the full length VPg, this halted replication of the MNV mutants was unexpected and striking. Such a dramatic phenotype warrants subsequent studies to investigate the involved molecular mechanism. Therefore, although the current study is based on MNV and may not be applicable to HuNV, these data may contribute to developing a novel target of therapeutic intervention to inhibit the replication of NoV.

## Accession Numbers

Atomic coordinates and structure factors for the RdRp-VPg(1-73)-RNA complex have been deposited in the Protein Data Bank (www.rcsb.org) under the identification code PDB ID 5Y3D.

## Author Contributions

KK, SH, MC, and HK conceived and analyzed the experimental data. J-HL, BP, IA, and JS solved the crystal structure. J-HL and SK conducted TEM. KH, J-HL, SB, and JC conducted the *in vitro* assay and cell-based *in vivo* assay. SH and KK wrote manuscript.

## Conflict of Interest Statement

The authors declare that the research was conducted in the absence of any commercial or financial relationships that could be construed as a potential conflict of interest.

## Footnotes

1 http://www.pymol.org/

## References

[B1] Adams P. D. Afonine P. V. Bunkoczi G. Chen V. B. Davis I. W. Echols N. (2010). PHENIX: a comprehensive Python-based system for macromolecular structure solution. *Acta Crystallogr. D Biol. Crystallogr.* 66(Pt 2) 213–221. 10.1107/S0907444909052925 20124702 PMC2815670

[B2] Alam I. Lee J. H. Cho K. J. Han K. R. Yang J. M. Chung M. S. (2012). Crystal structures of murine norovirus-1 RNA-dependent RNA polymerase in complex with 2-thiouridine or ribavirin. *Virology* 426 143–151. 10.1016/j.virol.2012.01.016 22341781

[B3] Bentham M. Holmes K. Forrest S. Rowlands D. J. Stonehouse N. J. (2012). Formation of higher-order foot-and-mouth disease virus 3D(Pol) complexes is dependent on elongation activity. *J. Virol.* 86 2371–2374. 10.1128/Jvi.05696-11 22156531 PMC3302376

[B4] Biering S. B. Choi J. Halstrom R. A. Brown H. M. Beatty W. L. Lee S. (2017). Viral replication complexes are targeted by LC3-guided interferon-inducible GTPases. *Cell Host Microbe* 22 74.e7–85.e7. 10.1016/j.chom.2017.06.005 28669671 PMC5591033

[B5] Bok K. Green K. Y. (2012). Norovirus gastroenteritis in immunocompromised patients. *N. Engl. J. Med.* 367 2126–2132. 10.1056/NEJMra1207742 23190223 PMC4944753

[B6] Brunger A. T. DeLaBarre B. Davies J. M. Weis W. I. (2009). X-ray structure determination at low resolution. *Acta Crystallogr. D Biol. Crystallogr.* 65 128–133. 10.1107/S0907444908043795 19171967 PMC2631637

[B7] Burroughs J. N. Brown F. (1978). Presence of a covalently linked protein on calicivirus RNA. *J. Gen. Virol.* 41 443–446. 10.1099/0022-1317-41-2-443 569187

[B8] Calvert J. G. Nagy E. Soler M. Dobos P. (1991). Characterization of the Vpg Dsrna linkage of infectious pancreatic necrosis virus. *J. Gen. Virol.* 72 2563–2567. 10.1099/0022-1317-72-10-2563 1919532

[B9] Chen C. Wang Y. Shan C. Sun Y. Xu P. Zhou H. (2013). Crystal structure of enterovirus 71 RNA-dependent RNA polymerase complexed with its protein primer VPg: implication for a trans mechanism of VPg uridylylation. *J. Virol.* 87 5755–5768. 10.1128/JVI.02733-12 23487447 PMC3648134

[B10] Chung L. Bailey D. Leen E. N. Emmott E. P. Chaudhry Y. Roberts L. O. (2014). Norovirus translation requires an interaction between the C terminus of the genome-linked viral protein VPg and eukaryotic translation initiation factor 4G. *J. Biol. Chem.* 289 21738–21750. 10.1074/jbc.M114.550657 24928504 PMC4118132

[B11] Emsley P. Cowtan K. (2004). Coot: model-building tools for molecular graphics. *Acta Crystallogr. D Biol. Crystallogr.* 60(Pt 1), 2126–2132. 10.1107/S0907444904019158 15572765

[B12] Ettayebi K. Crawford S. E. Murakami K. Broughman J. R. Karandikar U. Tenge V. R. (2016). Replication of human noroviruses in stem cell-derived human enteroids. *Science* 353 1387–1393. 10.1126/science.aaf5211 27562956 PMC5305121

[B13] Ferrer-Orta C. Arias A. Agudo R. Perez-Luque R. Escarmis C. Domingo E. (2006). The structure of a protein primer-polymerase complex in the initiation of genome replication. *EMBO J.* 25 880–888. 10.1038/sj.emboj.7600971 16456546 PMC1383552

[B14] Goodfellow I. (2011). The genome-linked protein VPg of vertebrate viruses - a multifaceted protein. *Curr. Opin. Virol.* 1 355–362. 10.1016/j.coviro.2011.09.003 22440837 PMC3541522

[B15] Green K. Y. (2007). “Caliciviruses: the noroviruses,” in *Fields Virology* 5th Edn eds Knipe P. M. H. D. M. Griffin D. E. Lamb R. A. Martin M. A. Roizman B. Straus S. E. (Philadelphia, PA: Wolters Kluwer Health) 949–979.

[B16] Gruez A. Selisko B. Roberts M. Bricogne G. Bussetta C. Jabafi I. (2008). The crystal structure of coxsackievirus B3 RNA-dependent RNA polymerase in complex with its protein primer VPg confirms the existence of a second VPg binding site on Picornaviridae polymerases. *J. Virol.* 82 9577–9590. 10.1128/Jvi.00631-08 18632861 PMC2546979

[B17] Han K. R. Alhatlani B. Y. Cho S. Lee J. H. Hosmillo M. Goodfellow I. G. (2017). Identification of amino acids within norovirus polymerase involved in RNA binding and viral replication. *J. Gen. Virol.* 98 1311–1315. 10.1099/jgv.0.000826 28640742

[B18] Han K. R. Choi Y. Min B. S. Jeong H. Cheon D. Kim J. (2010). Murine norovirus-1 3Dpol exhibits RNA-dependent RNA polymerase activity and nucleotidylylates on Tyr of the VPg. *J. Gen. Virol.* 91(Pt 7) 1713–1722. 10.1099/vir.0.020461-0 20219896

[B19] Hardy M. E. (2005). Norovirus protein structure and function. *FEMS Microbiol. Lett.* 253 1–8. 10.1016/j.femsle.2005.08.031 16168575

[B20] Herbert T. P. Brierley I. Brown T. D. (1997). Identification of a protein linked to the genomic and subgenomic mRNAs of feline calicivirus and its role in translation. *J. Gen. Virol.* 78(Pt 5) 1033–1040. 10.1099/0022-1317-78-5-1033 9152420

[B21] Hogbom M. Jager K. Robel I. Unge T. Rohayem J. (2009). The active form of the norovirus RNA-dependent RNA polymerase is a homodimer with cooperative activity. *J. Gen. Virol.* 90(Pt 2) 281–291. 10.1099/vir.0.005629-0 19141436

[B22] Hwang S. Maloney N. S. Bruinsma M. W. Goel G. Duan E. Zhang L. (2012). Nondegradative role of Atg5-Atg12/ Atg16L1 autophagy protein complex in antiviral activity of interferon gamma. *Cell Host Microbe* 11 397–409. 10.1016/j.chom.2012.03.002 22520467 PMC3348177

[B23] Jiang J. Laliberte J. F. (2011). The genome-linked protein VPg of plant viruses - a protein with many partners. *Curr. Opin. Virol.* 1 347–354. 10.1016/j.coviro.2011.09.010 22440836

[B24] Jones M. K. Watanabe M. Zhu S. Graves C. L. Keyes L. R. Grau K. R. (2014). Enteric bacteria promote human and mouse norovirus infection of B cells. *Science* 346 755–759. 10.1126/science.1257147 25378626 PMC4401463

[B25] Karst S. M. Wobus C. E. Lay M. Davidson J. Virgin H. W. (2003). STAT1-dependent innate immunity to a Norwalk-like virus. *Science* 299 1575–1578. 10.1126/science.1077905 12624267

[B26] Lee J.-H. Alam I. Han K. R. Cho S. Shin S. Kang S. (2011). Crystal structures of murine norovirus-1 RNA-dependent RNA polymerase. *J. Gen. Virol.* 92 1607–1616. 10.1099/vir.0.031104-0 21471315

[B27] Leen E. N. Kwok K. Y. Birtley J. R. Simpson P. J. Subba-Reddy C. V. Chaudhry Y. (2013). Structures of the compact helical core domains of feline calicivirus and murine norovirus VPg proteins. *J. Virol.* 87 5318–5330. 10.1128/JVI.03151-12 23487472 PMC3648151

[B28] McFadden N. Bailey D. Carrara G. Benson A. Chaudhry Y. Shortland A. (2011). Norovirus regulation of the innate immune response and apoptosis occurs via the product of the alternative open reading frame 4. *PLoS Pathog.* 7:e1002413. 10.1371/journal.ppat.1002413 22174679 PMC3234229

[B29] Orchard R. C. Wilen C. B. Doench J. G. Baldridge M. T. McCune B. T. Lee Y. C. (2016). Discovery of a proteinaceous cellular receptor for a norovirus. *Science* 353 933–936. 10.1126/science.aaf1220 27540007 PMC5484048

[B30] Otwinowski Z. Minor W. (1997). Processing of X-ray diffraction data collected in oscillation mode. *Methods Enzymol*. 276 307–326. 10.1016/S0076-6879(97)76066-X 27754618

[B31] Patel M. M. Widdowson M. A. Glass R. I. Akazawa K. Vinje J. Parashar U. D. (2008). Systematic literature review of role of noroviruses in sporadic gastroenteritis. *Emerg. Infect. Dis.* 14 1224–1231. 10.3201/eid1408.071114 18680645 PMC2600393

[B32] Paul A. V. van Boom J. H. Filippov D. Wimmer E. (1998). Protein-primed RNA synthesis by purified poliovirus RNA polymerase. *Nature* 393 280–284. 10.1038/30529 9607767

[B33] Prilusky J. Felder C. E. Zeev-Ben-Mordehai T. Rydberg E. H. Man O. Beckmann J. S. (2005). FoldIndex: a simple tool to predict whether a given protein sequence is intrinsically unfolded. *Bioinformatics* 21 3435–3438. 10.1093/bioinformatics/bti537 15955783

[B34] Rohayem J. Robel I. Jager K. Scheffler U. Rudolph W. (2006). Protein-primed and de novo initiation of RNA synthesis by norovirus 3Dpol. *J. Virol.* 80 7060–7069. 10.1128/JVI.02195-05 16809311 PMC1489054

[B35] Sosnovtsev S. V. Belliot G. Chang K. O. Prikhodko V. G. Thackray L. B. Wobus C. E. (2006). Cleavage map and proteolytic processing of the murine norovirus nonstructural polyprotein in infected cells. *J. Virol.* 80 7816–7831. 10.1128/JVI.00532-06 16873239 PMC1563789

[B36] Spagnolo J. F. Rossignol E. Bullitt E. Kirkegaard K. (2010). Enzymatic and nonenzymatic functions of viral RNA-dependent RNA polymerases within oligomeric arrays. *RNA* 16 382–393. 10.1261/rna.1955410 20051491 PMC2811667

[B37] Strong D. W. Thackray L. B. Smith T. J. Virgin H. W. (2012). Protruding domain of capsid protein is necessary and sufficient to determine murine norovirus replication and pathogenesis in vivo. *J. Virol.* 86 2950–2958. 10.1128/Jvi.07038-11 22258242 PMC3302348

[B38] Subba-Reddy C. V. Goodfellow I. Kao C. C. (2011). VPg-primed RNA synthesis of norovirus RNA-dependent RNA polymerases by using a novel cell-based assay. *J. Virol.* 85 13027–13037. 10.1128/Jvi.06191-11 21994457 PMC3233154

[B39] Tang G. Peng L. Baldwin P. R. Mann D. S. Jiang W. Rees I. (2007). EMAN2: an extensible image processing suite for electron microscopy. *J. Struct. Biol.* 157 38–46. 10.1016/j.jsb.2006.05.009 16859925

[B40] Taube S. Kolawole A. O. Hohne M. Wilkinson J. E. Handley S. A. Perry J. W. (2013). A mouse model for human norovirus. *mBio* 4:e00450-13. 10.1128/mBio.00450-13 23860770 PMC3735125

[B41] Thorne L. G. Goodfellow I. G. (2014). Norovirus gene expression and replication. *J. Gen. Virol.* 95(Pt 2), 278–291. 10.1099/vir.0.059634-0 24243731

[B42] van Heel M. Harauz G. Orlova E. V. Schmidt R. Schatz M. (1996). A new generation of the IMAGIC image processing system. *J. Struct. Biol.* 116 17–24. 10.1006/jsbi.1996.0004 8742718

[B43] Wobus C. E. Karst S. M. Thackray L. B. Chang K. O. Sosnovtsev S. V. Belliot G. (2004). Replication of Norovirus in cell culture reveals a tropism for dendritic cells and macrophages. *PLoS Biol.* 2:e432. 10.1371/journal.pbio.0020432 15562321 PMC532393

[B44] Wobus C. E. Thackray L. B. Virgin H. W. T. (2006). Murine norovirus: a model system to study norovirus biology and pathogenesis. *J. Virol.* 80 5104–5112. 10.1128/JVI.02346-05 16698991 PMC1472167

[B45] Zamyatkin D. F. Parra F. Alonso J. M. M. Harki D. A. Peterson B. R. Grochulski P. (2008). Structural insights into mechanisms of catalysis and inhibition in Norwalk virus polymerase. *J. Biol. Chem.* 283 7705–7712. 10.1074/jbc.M709563200 18184655

