# Corrigendum: Insight Into the Interaction Between RNA Polymerase and VPg for Murine Norovirus Replication

**DOI:** 10.3389/fmicb.2019.00032

**Published:** 2019-01-31

**Authors:** Ji-Hye Lee, Beom Seok Park, Intekhab Alam, Kang R. Han, Scott B. Biering, Soo J. Kim, Jayoung Choi, Jong H. Seok, Mi S. Chung, Ho M. Kim, Seungmin Hwang, Kyung H. Kim

**Affiliations:** ^1^Department of Biotechnology & Bioinformatics, Korea University, Sejong, South Korea; ^2^Department of Biomedical Laboratory Science, College of Health Science, Eulji University, Gyeonggi-do, South Korea; ^3^Committee on Microbiology, The University of Chicago, Chicago, IL, United States; ^4^Graduate School of Medical Science and Engineering, College of Life Science and Bioengineering, Korea Advanced Institute of Science and Technology, Daejeon, South Korea; ^5^Department of Pathology, The University of Chicago, Chicago, IL, United States; ^6^Department of Food and Nutrition, Duksung Women's University, Seoul, South Korea

**Keywords:** norovirus, RNA-dependent RNA polymerase, VPg, interaction, viral replication

In the original article Intekhab Alam was not indicated as having contributed equally to the article. The author list above has been updated to reflect this. The corrected Author Contributions Statement appears below.

“KK, SH, MC, and HK conceived and analyzed the experimental data. J-HL, BP, IA, and JS solved the crystal structure. J-HL and SK conducted TEM. KH, J-HL, SB, and JC conducted the *in vitro* assay and cell-based *in vivo* assay. SH and KK wrote manuscript.”

Additionally, the city name in affiliation 2 was incorrectly given as Daejeon. The correct city name is Gyeonggi-do.

The authors apologize for these errors and state that this does not change the scientific conclusions of the article in any way. The original article has been updated.

## Conflict of Interest Statement

The authors declare that the research was conducted in the absence of any commercial or financial relationships that could be construed as a potential conflict of interest.

