# Supplementary material for: Insight Into the Interaction Between RNA Polymerase and VPg for Murine Norovirus Replication
Source: Front Microbiol. 2018 Jul 3;9:1466. doi: 10.3389/fmicb.2018.01466 (PMC6046605; doi:10.3389/fmicb.2018.01466)
Supplement: Supplementary file 1 [file Presentation_1.pdf]

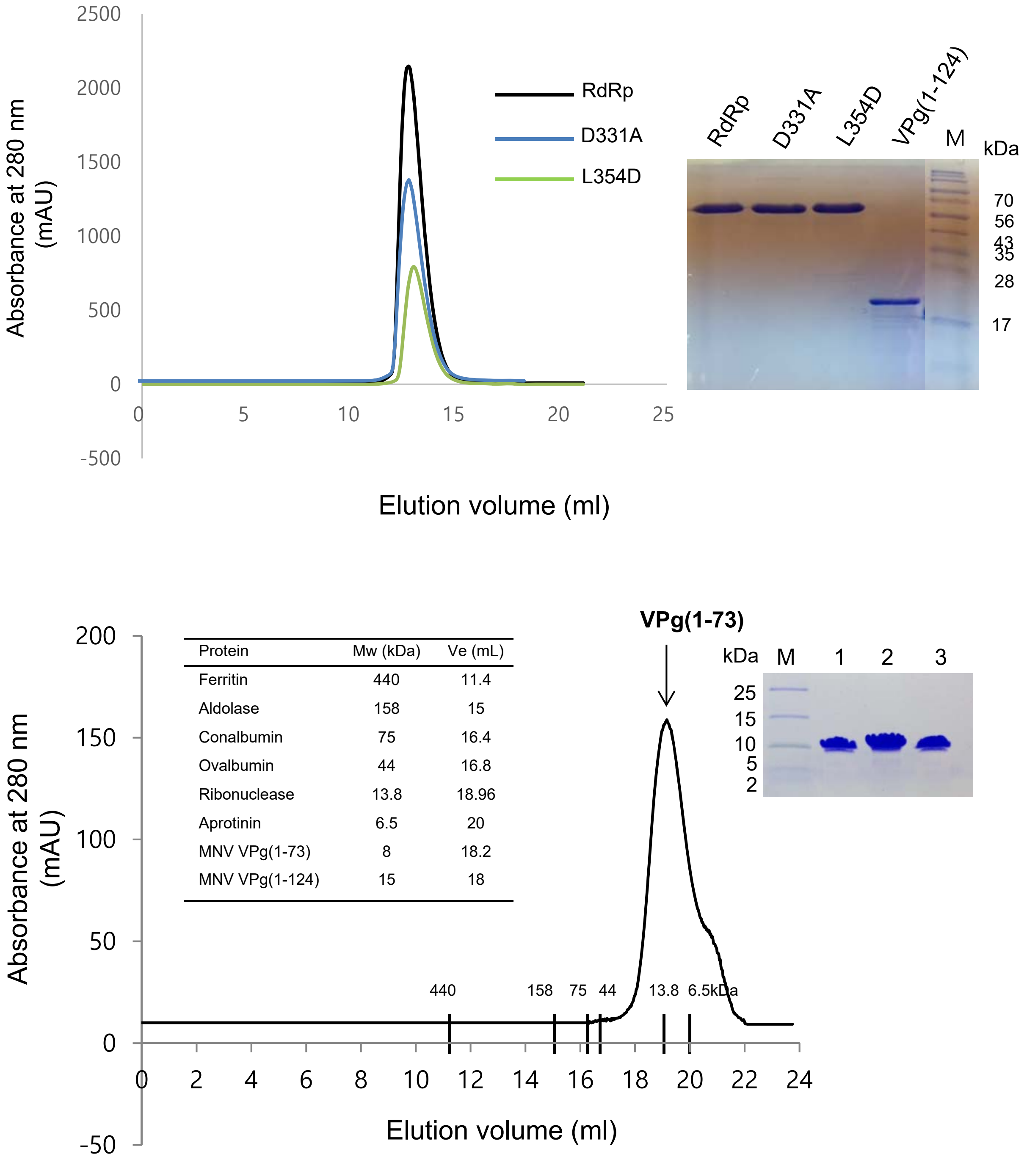

Protein	Mw (kDa)	Ve (mL)
Ferritin	440	11.4
Aldolase	158	15
Conalbumin	75	16.4
Ovalbumin	44	16.8
Ribonuclease	13.8	18.96
Aprotinin	6.5	20
MNV VPg(1-73)	8	18.2
MNV VPg(1-124)	15	18

Figure S1. Elution profiles of the MNV-1 RdRp and VPg(1-73) from gel filtration chromatography. RdRp native and mutants and VPg(1-124) were purifed using superdex 200 gel filtration chromatography and analysed by SDS-PAGE (upper panel), and VPg(1-73) was purifed using superdex 75 gel filtration chromatography and analysed by tricine-SDS-PAGE (inset) (lower panel).
