# Supplementary material for: Insight Into the Interaction Between RNA Polymerase and VPg for Murine Norovirus Replication
Source: Front Microbiol. 2018 Jul 3;9:1466. doi: 10.3389/fmicb.2018.01466 (PMC6046605; doi:10.3389/fmicb.2018.01466)
Supplement: Supplementary file 2 [file Presentation_2.pdf]

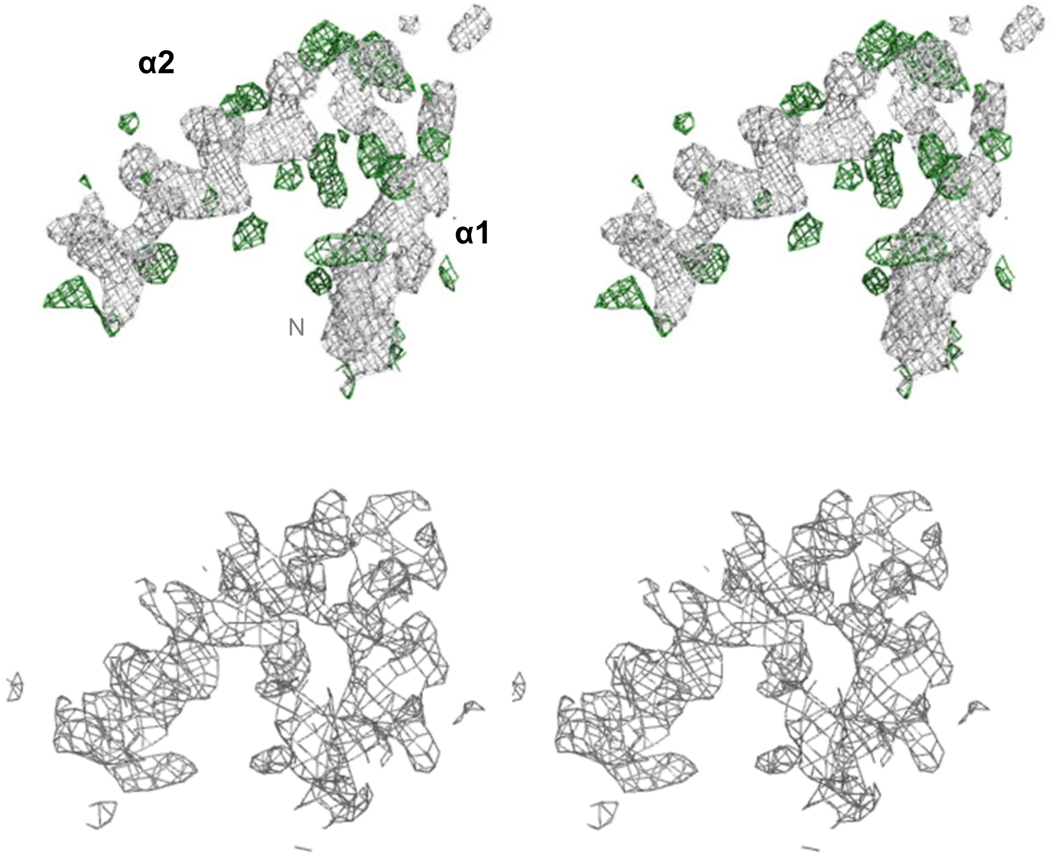

Figure S2. **Stereo view of the eletron density maps.** (Upper panel) the $2|F_o|-|F_c|$ electron density maps (0.7 σ in gray maps) with the positive $|F_o|-|F_c|$ electron density maps (red density) and simulated annealing omit maps (1.2 σ in gray maps) showing unbiased electron densities (lower panel) for VPg in the RdRp-VPg(1-73) complex.
