# Supplementary material for: Insight Into the Interaction Between RNA Polymerase and VPg for Murine Norovirus Replication
Source: Front Microbiol. 2018 Jul 3;9:1466. doi: 10.3389/fmicb.2018.01466 (PMC6046605; doi:10.3389/fmicb.2018.01466)
Supplement: Supplementary file 3 [file Presentation_3.pdf]

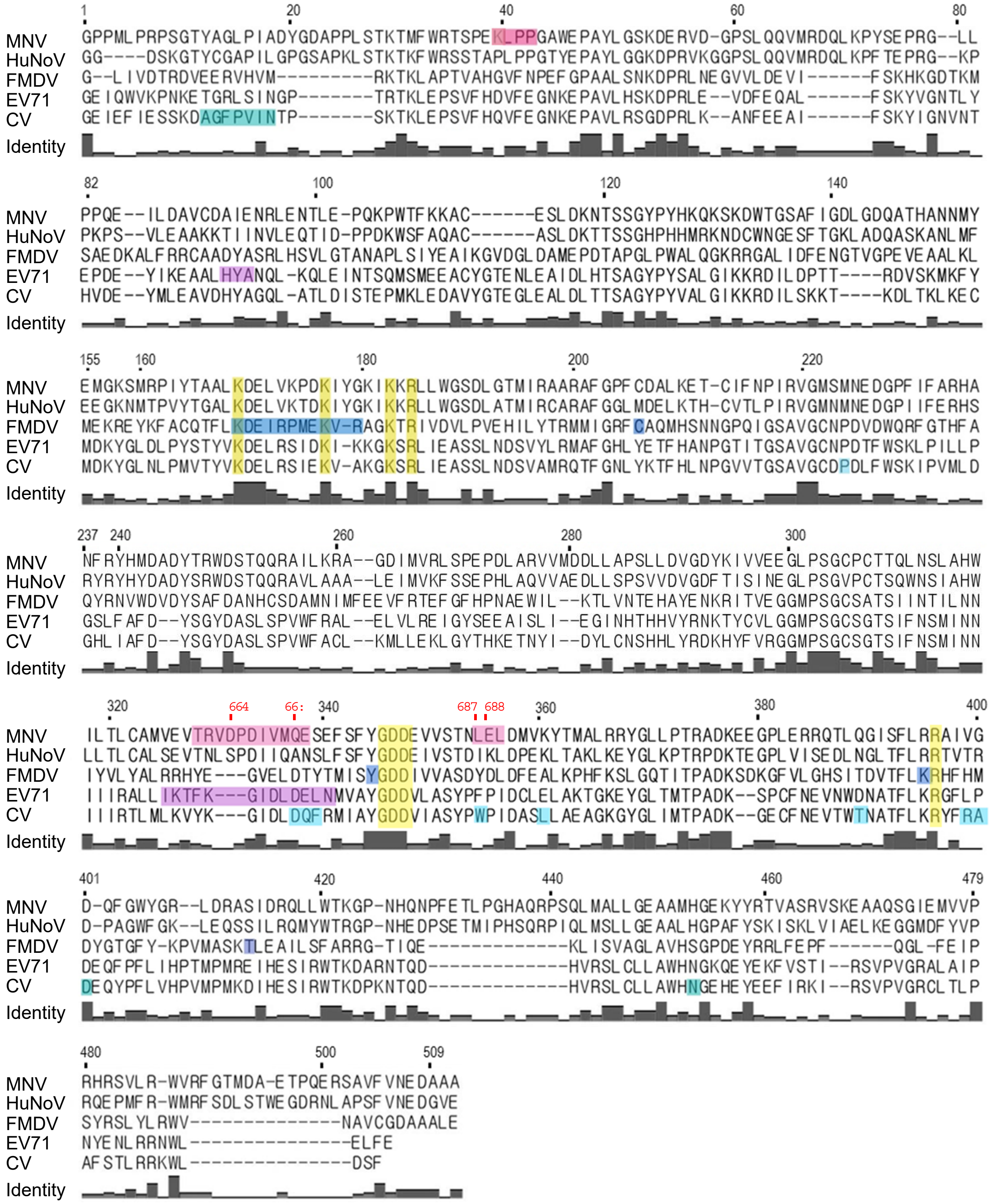

Figure S3. Sequence alignment of RdRp molecules. Sequence alignments of RdRp from MNV, HuNV, FMDV, EV71, and CV are shown. Above the alignment, amino acid sequences are presented in blocks of 20 amino acids, and histograms below the alignment indicate the conservation level of amino acids. The catalytic residues GDD at the active site, involved in RNA binding are highlighted in yellow, and the residues involved in VPg binding are highlighted in the same color code as in Fig. 2C: MNV-1, EV71, FMDV, and CV in red, pink, blue, and cyan color, respectively.
