# Supplementary material for: Insight Into the Interaction Between RNA Polymerase and VPg for Murine Norovirus Replication
Source: Front Microbiol. 2018 Jul 3;9:1466. doi: 10.3389/fmicb.2018.01466 (PMC6046605; doi:10.3389/fmicb.2018.01466)
Supplement: Supplementary file 4 [file Presentation_4.pdf]

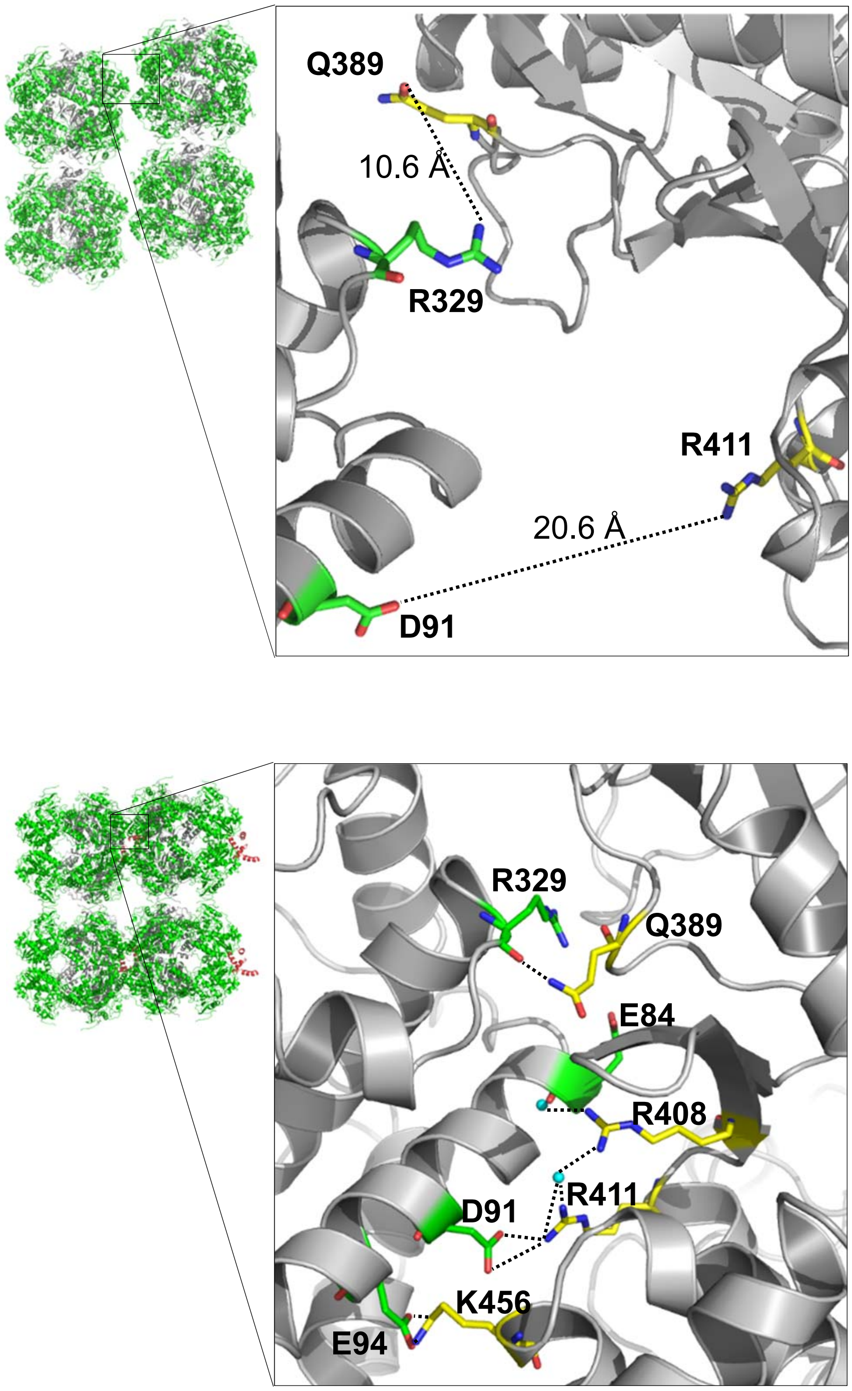

Figure S4. Comparison of the interactions between RdRp and RdRp in the native and RdRp-VPg(1-73) complex structures. The interactions between Arg329 and Gln389 and between Asp91 and Arg411 of adjacent RdRp molecules are absent in the native (upper panel), whereas they are distinctly different in the RdRp-VPg(1-73) complex structure (lower panel).
