# Supplementary material for: Insight Into the Interaction Between RNA Polymerase and VPg for Murine Norovirus Replication
Source: Front Microbiol. 2018 Jul 3;9:1466. doi: 10.3389/fmicb.2018.01466 (PMC6046605; doi:10.3389/fmicb.2018.01466)
Supplement: Supplementary file 5 [file Presentation_5.pdf]

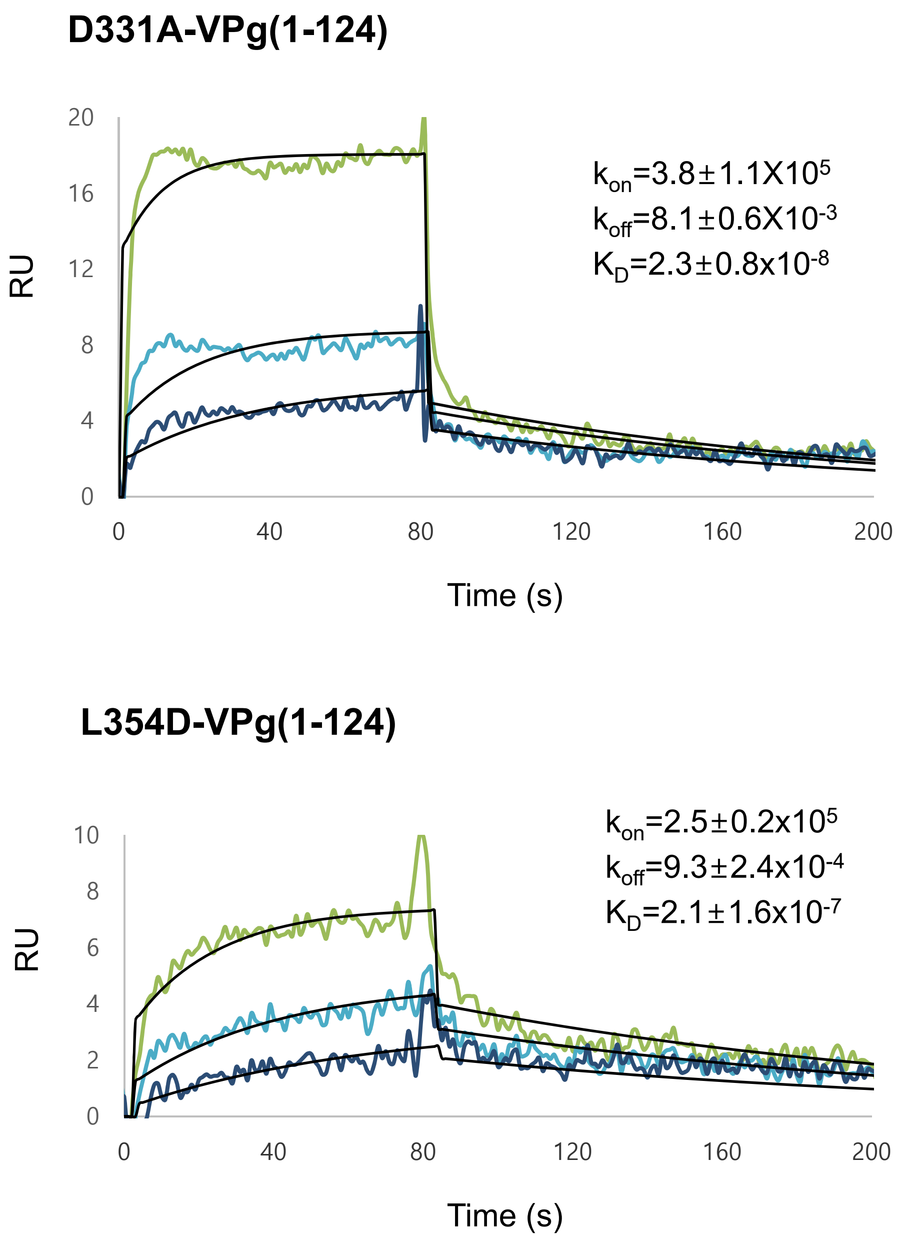

Figure S5. SPR binding assay. The SPR sensorgram to measure the affinity of VPg to RdRp D331A and L354D. The binding affinity constant (K_D) was determined from the association (k_{on}) and dissociation (k_{off}) rates by evaluating the 1:1 Langmuir binding model kinetics in the sensorgrams. Colored curves depict experimental data at different analyte concentrations and fitted curves modelled to describe a 1:1 binding event are overlaid in black. The experiments were performed in duplicates.
